# Snf1 cooperates with the CWI MAPK pathway to mediate the degradation of Med13 following oxidative stress

**DOI:** 10.15698/mic2018.08.641

**Published:** 2018-06-25

**Authors:** Stephen D. Willis, David C. Stieg, Kai Li Ong, Ravina Shah, Alexandra K. Strich, Julianne H. Grose, Katrina F. Cooper

**Affiliations:** 1 Department of Molecular Biology, Graduate School of Biomedical Sciences, Rowan University, Stratford, NJ, 08084, USA.; 2 Department of Microbiology and Molecular Biology, Brigham Young University, Provo, UT 84602, USA.; 3 Current address: Department of Biological Sciences, Rowan University, 201 Mullica Hill Rd, Glassboro, NJ 08028. USA.; 4 Current address: Shawnee High School, Medford, New Jersey 08055, USA.

**Keywords:** cyclin C, Cdk8, Med13, SCF^Grr1^, AMPK, Snf1, ubiquitin mediated destruction, signal transduction, H_2_O_2_ stress, MAPK

## Abstract

Eukaryotic cells, when faced with unfavorable environmental conditions, mount either pro-survival or pro-death programs. The conserved cyclin C-Cdk8 kinase plays a key role in this decision. Both are members of the Cdk8 kinase module that, along with Med12 and Med13, associate with the core Mediator complex of RNA polymerase II. In *Saccharomyces cerevisiae,* oxidative stress triggers Med13 destruction, which releases cyclin C into the cytoplasm to promote mitochondrial fission and programmed cell death. The SCF^Grr1^ ubiquitin ligase mediates Med13 degradation dependent on the cell wall integrity pathway, MAPK Slt2. Here we show that the AMP kinase Snf1 activates a second SCF^Grr1^ responsive degron in Med13. Deletion of Snf1 resulted in nuclear retention of cyclin C and failure to induce mitochondrial fragmentation. This degron was able to confer oxidative-stress-induced destruction when fused to a heterologous protein in a Snf1 dependent manner. Although *snf1*∆ mutants failed to destroy Med13, deleting the degron did not prevent destruction. These results indicate that the control of Med13 degradation following H_2_O_2_ stress is complex, being controlled simultaneously by CWI and MAPK pathways.

## INTRODUCTION

All eukaryotic cells are continually exposed to changing environmental conditions. Consequently, they have evolved elaborate mechanisms to both sense damage and transmit this signal to the nucleus. The resulting response varies dependent upon the stress encountered but in gross terms cells have to decide whether to activate pro-survival or pro-death programs. Despite this being a critical decision point, what remains understudied are the molecular details of how cells decide their fate upon encountering unfavorable environments. Previous studies revealed that the conserved cyclin C protein plays a key role in this decision in response to increased environmental reactive oxygen species (ROS) 1,2,3,4,5,6,7,8,9. Cyclin C, together with its kinase partner Cdk8, Med12 and Med13, form the Cdk8 Kinase Module (CKM) of the multi-subunit Mediator complex. This complex acts as an interface between DNA bound transcription factors and RNA polymerase II (RNAP-II 10,11,12). When the CKM module is bound, it predominantly negatively regulates expression of a subset of stress response genes 1,13,14,15,16. Following an increase in environmental ROS (induced by H_2_O_2_ treatment), this repression is relieved by the nuclear release of cyclin C to the cytoplasm 4,7,8. Intriguingly, here cyclin C plays a second role directing stress-induced mitochondrial fission as well as promoting programmed cell death (PCD) 7,8. Consistent with this function, cells lacking cyclin C are less able to execute stress-induced mitochondria fission and are more resistant to oxidative stress 3,7. Taken together, these results argue that cyclin C nuclear release must be carefully controlled as it signals a commitment to PCD.

**Figure 1:**
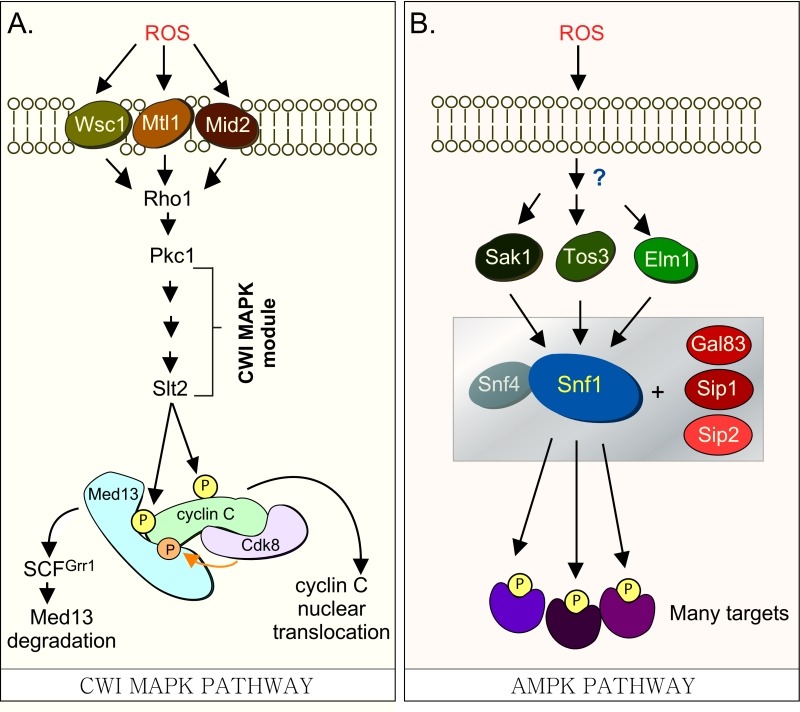
FIGURE 1: **(A)** **Cdk8 module regulation by the CWI MAPK pathway. **H_2_O_2 _stimulates cell wall sensors Wsc1, Mtl1 and Mid2, leading to activation of Rho1 that in turn triggers the cell wall integrity (CWI) MAPK pathway by activating Protein Kinase C (Pkc1). Activation of this cascade triggers the MAPK, Slt2, to directly phosphorylate cyclin C, an event required for the 1^st^ step towards its release from the nucleus. The second step requires Slt2 to directly phosphorylate Med13-degron^742-844^, which targets it for ubiquitin mediated degradation by SCF^Grr1^. Cyclin C-Cdk8 activity is needed to prime the degron before it is recognized by SCF^Grr1^ 9. **(B) Outline of the AMPK pathway in yeast.** It remains unknown how this pathway is activated in response to H_2_O_2_ stress. The gray box represents the Snf1 kinase complex (see text for details).

Our previous studies have revealed that a complex molecular mechanism controls cyclin C nuclear release. In response to environmental ROS, cyclin C is directly phosphorylated by Slt2, the MAP kinase of the Cell Wall Integrity (CWI) signal transduction pathway (6 and Fig. 1A). This canonical pathway is characterized by a family of cell-surface sensors (Wsc1, Mid2 and Mtl1 17) that transmit the stress to a small G protein Rho1, which thereafter activates protein kinase C (Pkc1 18,19). Once activated, Pkc1 transmits the intracellular signal to the MAPK Slt2/Mpk1 20 as well as to the pseudo-kinase Kdx1/Mlp1 via the MAPK module 21. In addition to cyclin C 6, Slt2 directly phosphorylates two other transcription factors (Rlm1, Swi4/Swi6) that stimulate the expression of other stress response genes 21,22,23,24. More recently, Slt2 has also been shown to regulate gene expression by phosphorylating tyrosine-1 of the RNAP II carboxy-terminal domain 25. This event is associated with pause/termination processes in mammals 26. Intriguingly, it is required for the loss of cyclin C and Cdk8 from target genes following stress in yeast 25.

**Figure 2:**
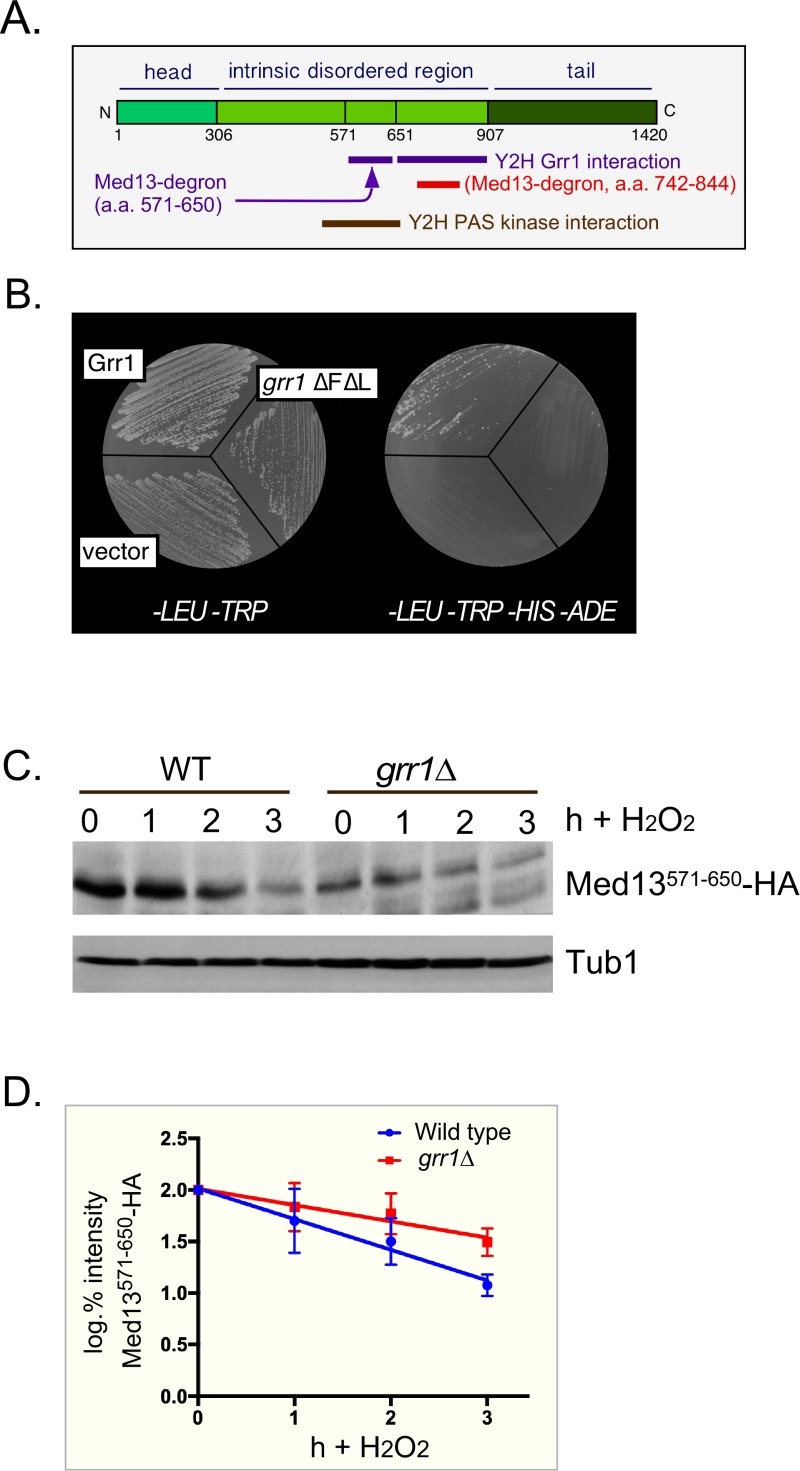
FIGURE 2: Med13 contains two H_2_O_2_ stress responsive degrons. **(A)** Cartoon of the results from ProteinPredict^®^ 89 analysis of yeast Med13. The amino and carboxyl terminal domains are structured and separated by a large intrinsic disordered region. The positions of the two degrons are indicated. **(B)** Yeast two hybrid analysis of degron^571-650 ^and Grr1 derivatives. Yeast PJ69-4a cells harboring Med13-activating domain plasmid (pDS15) and either pAS2, pAS-Grr1 or pAS2-Grr1∆F∆L binding domain plasmids were grown on *-LEU, -TRP *drop out medium to select for both plasmids (left panel) and *-TRP, -LEU, -HIS -ADE* (right panel) to test for Med13-Grr1 interaction. **(C)** Wild-type (RSY10) or *grr1*∆ cells (RSY1770) harboring degron^571-650 ^(pDS15) were treated with 0.4 mM H_2_O_2_ for the timepoints indicated and Med13^571-650^-HA levels analyzed by Western blot. Tub1 levels were used as loading controls. **(D)** Degradation kinetics of the degron^571-650 ^constructs shown in C. Values represent averages ± SD from a total of at least two Western blots from two independent experiments.

In *S. cerevisiae*, Med13 destruction is required for cyclin C nuclear release 27. Consistent with this, cyclin C is cytoplasmic in unstressed *med13*∆ and the mitochondria are predominantly fragmented 27. More recently we identified SCF^Grr1^ as the E3 ligase that mediates Med13 destruction 9. Like other SCF targets 28, recognition of this phospho-degron requires it first to be primed by one kinase (cyclin C-Cdk8) and then activated by another (Slt2). These studies also revealed that phosphorylation of cyclin C by Slt2 is required for the SCF^Grr1^ to recognize Med13 (9 and Fig. 1A). Like the Slt2-degron, this domain lies within the large intrinsic disordered region (IDR) of Med13 (Fig. 2A). IDR’s are defined by a continuous stretch of disordered promoting residues which can easily transition into more ordered states. These structural transitions afford IDR’s great flexibility and as such these regions are known to play key roles in macro-molecular decisions especially those that influence signaling pathways 29,30.

In addition to Slt2, the highly conserved 5’ adenosine monophosphate-activated protein kinase (AMPK), which plays a major role in the utilization of alternative carbon sources after glucose depletion 32, is also activated in response to various environmental stresses including oxidative stress 33. In *S. cerevisiae*, the catalytic subunit of the heterotrimeric AMPK complex is encoded by *SNF1*. Other members of the complex (outlined in Fig. 1B) include two regulatory subunits, the γ subunit Snf4 and one of the three alternative β subunits, Sip1, Sip2, or Gal83 32. The three β isoforms determine the respective cellular addresses after activation of Snf1, with the Snf1-Gal83 isoform being enriched in the nucleus 34,35,36. The catalytic activity of Snf1 is regulated by phosphorylation at Thr-210, which is located in the activation loop of its kinase domain 37. This is executed by one of three upstream kinases, Sak1, Tos3, or Elm1 33,38,39. These, in turn, are activated by an unknown mechanism in response to a variety of stresses, which lends specificity to the system 33.

In this report we show that Snf1, Sak1 and at least one β isoform are required for the H_2_O_2_ induced degradation of Med13. Using yeast two-hybrid analysis, the Snf1-interacting domain on Med13 was identified. This domain lies in the large IDR of Med13 and is recognized by SCF^Grr1 ^after Snf1 directed phosphorylation. Consistent with this, Snf1 is required for efficient cyclin C nuclear release following H_2_O_2_ stress. Taken together, this reveals that Med13 degradation is regulated by two SCF^Grr1^ degrons that are regulated by three different classes of kinases, a Cdk, a MAPK and an AMPK. As all three kinases are required for Med13 degradation, this complex molecular mechanism ensures that cyclin C nuclear release is tightly controlled and prevents its untimely release into the cytoplasm.

## RESULTS

### Med13 contains two SCF^Grr1 ^phospho-degrons

We have previously shown that SCF^Grr1^ is the E3 ligase responsible for mediating Med13 degradation following H_2_O_2_ stress 9. This degron (amino acids 742-844, Fig. 2A,) is primed by cyclin C-Cdk8 and activated by Slt2. In these studies we also observed that another Med13 domain (amino acids 571-650) can also bind to Grr1 using the Gal4 yeast two hybrid (Y2H) assay 40. These results were repeated using two baits, wild-type Grr1 and a *grr1∆F*∆*L* mutant, which can neither bind to the SCF or recognize substrates 41,42. As anticipated, the Gal4 activating domain Med13^571-650 ^fusion protein (hereon out referred to as degron^571-650^) can associate with wild-type Grr1 as an interaction is detected selecting for the dual *HIS3* and *ADE2* reporter genes (Fig. 2B). The vector control and mutant bait were unable to grow on this media suggesting that the interaction is specific.

To confirm that degron^571-650 ^is responsive to SCF^Grr1^ we examined its degradation, via Western blot analysis, following 0.4 mM H_2_O_2_ treatment in wild-type and *grr1*∆ cells. This concentration of H_2_O_2_ induces mitochondrial outer membrane permeabilization (MOMP) dependent regulated cell death response in yeast 43,44,45 and triggers cyclin C nuclear release 4,6,7,27. The results show that although less degron^571-650 ^is present in unstressed *grr1*∆ cells, the protein is more stable following H_2_O_2_ stress (Fig. 2C and quantified in 2D). Taken together with the Y2H data, these results argue that degron^571-650 ^is a SCF^Grr1^ responsive degron. They are also consistent with our previous published work demonstrating that Grr1 is required for H_2_O_2_ induced degradation of Med13 9.

### Snf1 is required for stress-induced degradation of Med13

We next addressed if the H_2_O_2_ mediated destruction of degron^571-650 ^required Cdk8 kinase activity. To execute this, the degradation of degron^571-650 ^was monitored as described above, in cells deleted for cyclin C harboring either functional myc-tagged cyclin C or a vector control. The results revealed that degron^571-650 ^is degraded with the same kinetics in the presence or absence of cyclin C (Fig. S1A and quantified in Fig. S1B). These results suggest that another kinase phosphorylates degron^571-650^. One candidate is the PAS kinase. This kinase is required for glucose homeostasis, and is encoded by two orthologs Psk1 and Psk2 46. Intriguingly, previous Y2H studies have revealed that a domain of Med13 (amino acids 505-703), that encompasses degron^571-650^, interacts with Psk1 47. The Y2H assay was repeated using Psk1 as bait and the smaller degron^571-650 ^as prey and again an interaction was observed (Fig. S2A). We next tested if the PAS kinase plays a role in the degradation of full length Med13. Wild-type and *psk1*∆* psk2*∆ cells harboring a functional HA-tagged Med13 plasmid 9 were treated with H_2_O_2 _and Med13 degradation was monitored by Western blot analysis. The results show that Med13 is degraded with similar kinetics in the mutant and wild-type cells (Fig. S2B and quantified in Fig. S2C). These results indicate that although the PAS kinase can interact with degron^571-650^, it is not required for Med13 degradation following H_2_O_2_ stress.

**Figure 3:**
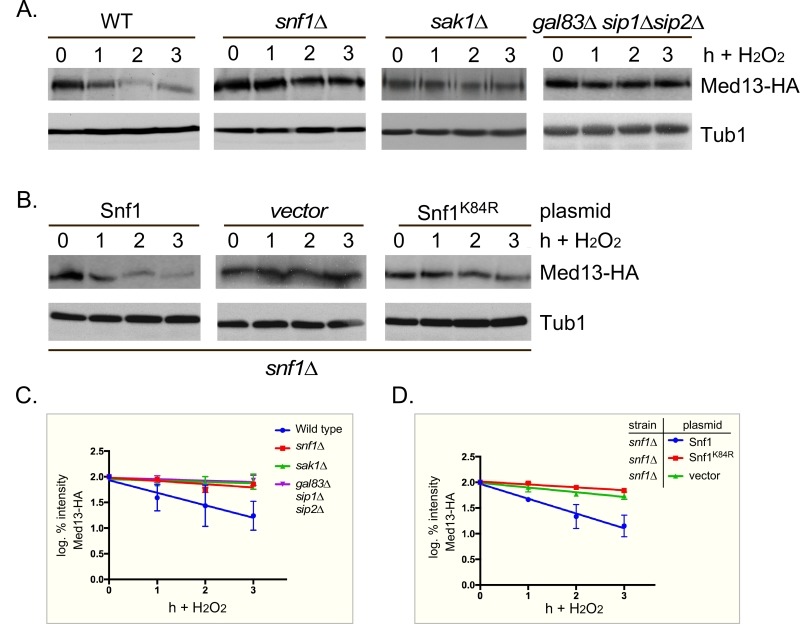
FIGURE 3: Snf1, Sak1 and at least one β subunit are required for degradation of Med13 following H_2_O_2_ stress. **(A)** Wild-type (RSY10), *snf1*∆ (RSY2080), *sak1*∆ (YPDahl17) and *gal83*∆* sip1*∆* sip2*∆ (MSY557) cells harboring full length Med13-HA (pKC801) were treated with 0.4 mM H_2_O_2_ for the timepoints indicated and Med13-HA levels analyzed by Western blot. Tub1 levels were used as a loading control. **(B)** *snf1*∆ cells harboring Med13-HA (pKC803) and either wild- type Snf1 (JG1193), a vector control (pRS316) or *snf1*^K84R^ (JG1338) were treated and analyzed as described in A. **(C and D)** Degradation kinetics of the Med13-HA shown in A and B. Values represent averages ± SD from a total of at least two Western blots from independent experiments.

PAS kinase activation by carbon sources is dependent on the Snf1 complex 48,49. Since Snf1 has many targets 50, we next tested if Snf1 mediates Med13 degradation following H_2_O_2_ stress as just described. The results show that Med13 is significantly more stable in *snf1*∆ cells (Fig. 3A and quantified in Fig. 3C). Similar results were obtained when the Snf1 kinase dead mutant (K84R, 51) was the only source of Snf1 (Fig. 3B and quantified in Fig. 3D). Taken together, these results indicate that Snf1 activity is required for Med13 degradation following H_2_O_2_ stress.

Sak1 has been identified as the AMPK kinase that is activated in response to oxidative stress 33. Therefore, we next addressed if Sak1 is required for H_2_O_2_ induced Med13 degradation. The degradation assays described above were repeated in *sak1*∆ cells and results revealed that Med13 was again significantly more stable in *sak1*∆ than wild-type cells (Fig. 3A and quantified in Fig. 3C). This result supports the previously proposed model that Sak1 is the AMPKK that activates Snf1 in response to oxidative stress 33. We next addressed if the nuclear enriched isoform, Snf1-Gal83, 34,35,36,52, is required for Med13 degradation under similar circumstances. Unexpectedly, the results show that Med13 is still degraded in *gal83*∆ cells (Fig. S3A). Likewise, similar results were obtained when Med13 degradation was monitored in a *sip1*∆* sip2*∆ strain (Fig. S3A). However, deletion of all three β subunits significantly inhibited the degradation of Med13 to a similar extend as observed in *snf1*∆ (Fig. 3A and quantified in Fig. 3C). Taken together, this suggests at least one of the β subunits of the Snf1 complex is required for Med13 degradation following H_2_O_2_ stress.

### Snf1 activation alone is not sufficient to mediate Med13 degradation

**Figure 4:**
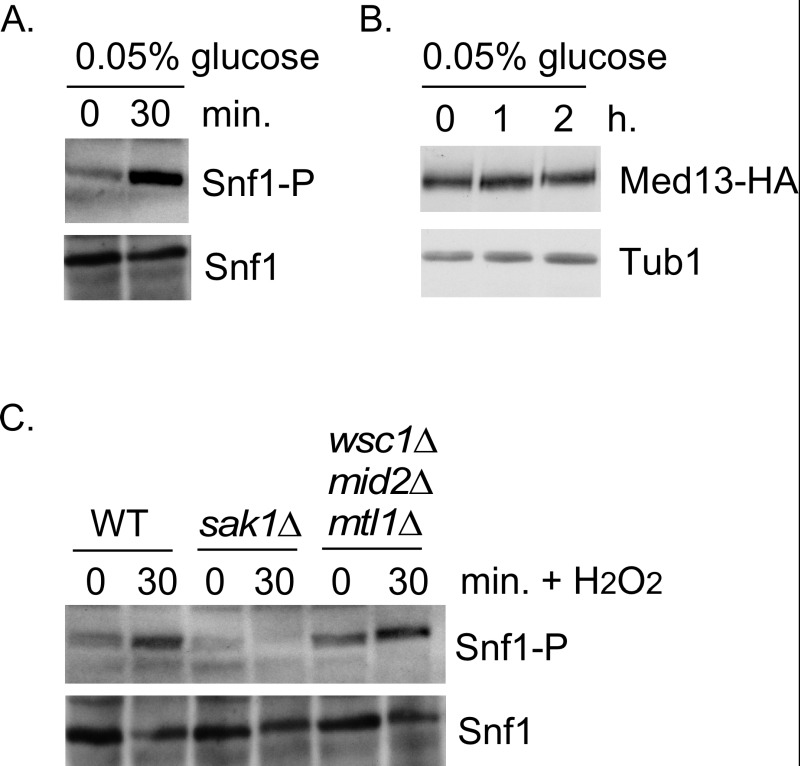
FIGURE 4: Snf1 activation does not mediate Med13 degradation. **(A)** Mid-log -type cells (RSY10) growing in 2% glucose (T=0) were switched to media containing 0.05% glucose. Snf1 phosphorylation and Snf1 itself were detected as described in materials and methods for the timepoint indicate. **(B)** Wild-type cells harboring Med13-HA (pKC801) were switched to media containing 0.05% glucose. Med13-HA levels were detected by Western blot analysis for the timepoints indicated. Tub1 was used as a loading control. **(C)** As in (A) except that Snf1 phosphorylation was monitored after 0.4 mM H_2_O_2_ treatment in the strains indicated.

We next addressed if Snf1 activation was sufficient to mediate Med13 degradation in the absence of H_2_O_2_ stress. To execute this Med13 levels were examined in wild-type cells after they had been switched from 2% to 0.05% glucose which, is sufficient to activate Snf1 (Fig. 4A and 33). No differences in Med13 levels were observed following glucose deprivation (Fig. 4B). Taken with the results presented in Fig. 3, this suggests that Snf1 activation is necessary but not sufficient to mediate the degradation of Med13 following H_2_O_2_ treatment.

### The CWI pathway sensors proteins are not required for Snf1 activation

It is known that both the CWI and AMPK pathways are activated in response to oxidative stress, but how these two pathways communicate is not well understood 53. Importantly, although it is known how the signal is transmitted to the CWI pathway, it is unclear how the AMPK pathway is activated (Fig. 1B). One possibility is that the pathways crosstalk through the cell wall sensor proteins. To test this, we examined Snf1 phosphorylation status in a strain lacking the three cell wall sensors (Wsc1, Mid2 and Mtl1, 17). Consistent with previously published work 33, Snf1 is phosphorylated following H_2_O_2_ treatment in a Sak1 dependent manner (Fig. 4C). Likewise the cell wall sensor triple mutant is also able to activate Snf1 following H_2_O_2_ treatment (Fig. 4C). These results suggest that the activation of the CWI pathway is not required to transmit the stress signal to the AMPK pathway. Others have demonstrated that Snf1 is not required for activation of Slt2 54. Consistent with this, we observed that the Med13 degron^571-650 ^is still degraded following H_2_O_2_ stress when a mutant of cyclin C, that cannot be phosphorylated by Slt2 (S266A, 6), is used as the sole source of cyclin C (Fig. S1C and D). Taken together, these results argue that the AMPK and MAPK signaling pathways act independently of each other. The extension of this conclusion is that both pathways contribute independently to regulating Med13 destruction following H_2_O_2_ stress.

### The Snf1 degron lies within the IDR of Med13

**Figure 5:**
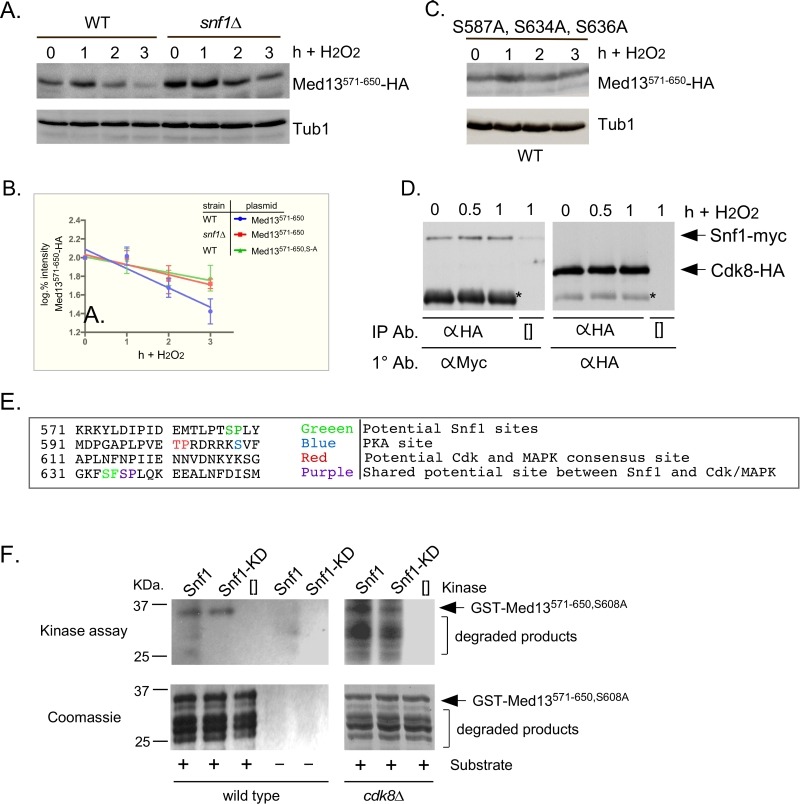
FIGURE 5: Snf1 phosphorylates degron^571-650^. **(A)** Mid-log wild-type (RSY10) or *snf1*∆ cultures (RSY202) harboring degron^571-650 ^(pDS15) were subjected to an H_2_O_2_ timecourse experiment and protein extracts analyzed by Western blot. Tub1 levels were used as loading controls. **(B) **Degradation kinetics of degron^571-650 ^shown in (A) and (C). Values represent averages ± SD from a total of at three Western blots from independent experiments. **(C)** As in (A) except that degron^571-650,S587A, S634A, S636A^ (pDS56) was analyzed in wild-type cells. **(D)** Co-immunoprecipitation analysis of Snf1-myc and Cdk8-HA. Mid log wild-type cells harboring Snf1-myc and Cdk8-HA on single copy plasmids were treated with 0.4 mM H_2_O_2_ for the timepoints shown. Protein extracts were immunoprecipitated with anti-HA, separated by SDS-PAGE Western analysis and the membrane probed with the antibodies shown. [] represents no IP antibody and the asterisk represents the heavy chain. See Fig. S3C for vector control. **(E) **Potential phospho-sites in Med13^571-650^. **(F)** Upper panels: Kinase assays using Snf1 and Snf1^K84R^-myc (kinase dead) immunoprecipitated from yeast protein extracts prepared from either wild type (left panel) or *cdk8*∆ cells (right panel) and Med13-degron^571-650 ^(GST-Med13^571-906,S608A ^purified from *E. coli*) as the substrate. The reactions were separated by SDS PAGE and subject to autoradiography. Lower panels: Coomassie stained gels showing the input used in the kinase assays.

**Figure 6:**
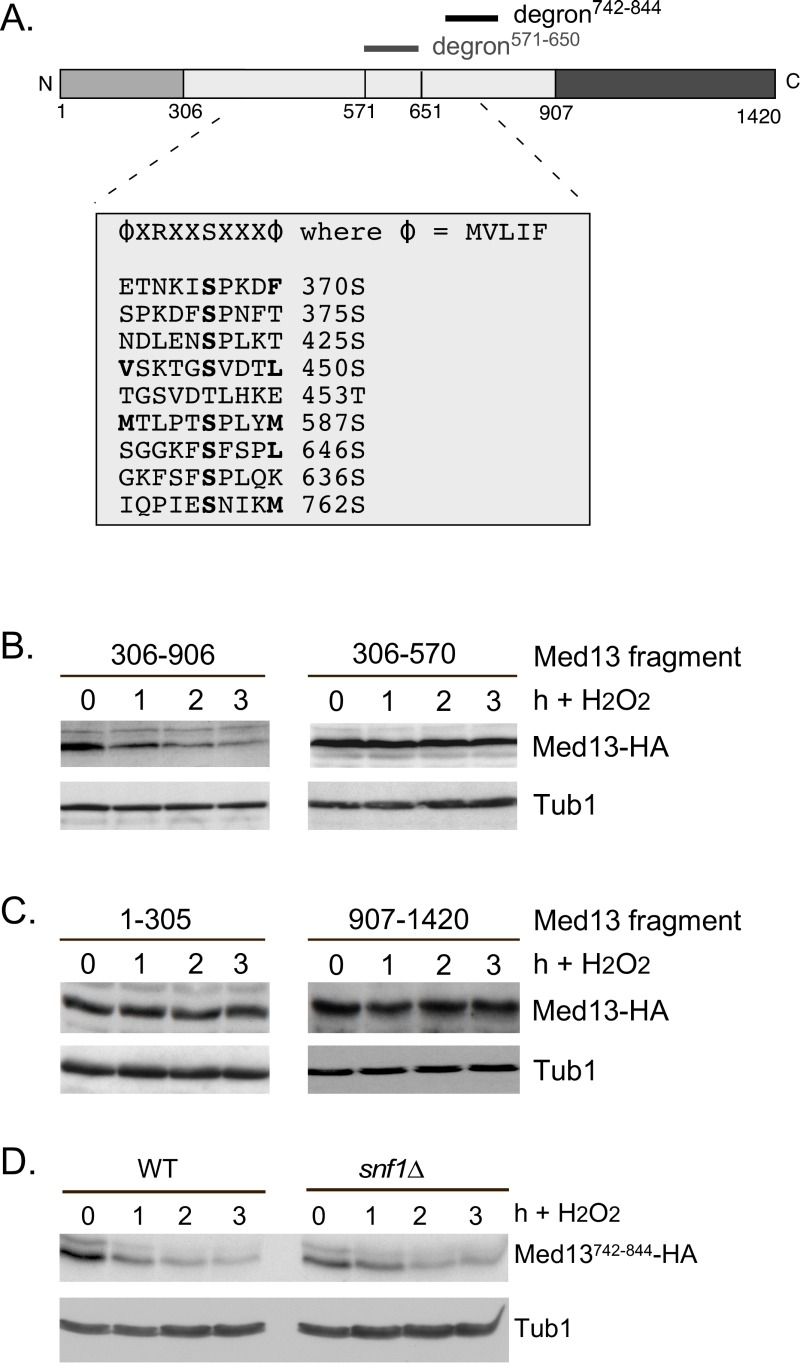
FIGURE 6: Other potential Snf1 sites in Med13 are not required for its degradation following H_2_O_2 _stress. **(A)** Map of Med13 outlying the positions of the two Med13 degrons, the consensus Snf1 target site 57 and potential Snf1 sites, identified by published proteomic screens. **(B)** and **(C)** Wild-type (RSY10) cultures harboring the NLS-Med13-HA constructs shown were grown to mid-log phase (0 h) then treated with 0.4 mM H_2_O_2 _for the indicated times. Med13-HA levels were determined by Western blot analysis. Tub1 levels were used as a loading control. **(D)** Mid-log wild type or *snf1*∆ cultures (RSY202) harboring HA tagged Med13 degron742-844 (pDS32) were subjected to an H_2_O_2_ timecourse experiment and protein extracts analyzed by Western blot. Tub1 levels were used as loading controls.

Snf1 has previously been shown to phosphorylate a SCF^Grr1^ phospho-degron in Pfk27, a key regulator of glycolysis 55. Taking this into account, we asked if the Gal4 activating domain Med13^571-650 ^fusion protein (degron^571-650^) is a Snf1 responsive degron by monitoring its H_2_O_2_ mediated degradation *snf1*∆ cells. The results show that degron^571-650 ^is more stable in *snf1*∆ cells compared to wild type following H_2_O_2_ stress (Fig. 5A and quantified in Fig. 5B). This suggests that degron^571-650 ^is a Snf1 responsive degron. To further support this conclusion, we examined the degradation kinetics of a degron mutated for either of the potential Snf1 phosphorylation sites previously identified in proteomic screens (S587 56 and S636 57, Fig.6A), although none of these sites perfectly fit the Snf1 consensus sequence 58,59. The studies revealed that both mutants were degraded following H_2_O_2_ treatment with kinetics similar to wild type (Fig. S3B). We also noted that serine 634 could potentially be phosphorylated by Snf1 (see Fig. 6A). As it lies very close to serine 636, we made a double mutant (S634A, S636A) and repeated the assay. Again this mutant degron was degraded following H_2_O_2 _treatment (Fig. S3B). However, the triple mutant (S587A, S634A, S636A) exhibited enhanced stability following H_2_O_2_ stress (Fig. 5C and quantitated in Fig. 5B). Taken together, these results suggest that Snf1 phosphorylates Med13 following H_2_O_2_ stress targeting S587, S634 and S636. Consistent with this model, we (Fig. 5D and S3C) and others 60 have shown that Snf1 can co-immunoprecipitate with Cdk8 both before and after H_2_O_2_ stress. As cyclin C directly binds to the adja-cent degron^742-844 ^(Fig. 2A and 9), this places Snf1 in close proximity to degron^571-650^. In addition, these data also support the notion suggested by others, that a subpopulation of Snf1 is nuclear in unstressed cells 34,52,60.

To address if Snf1 directly phosphorylates Med13, kinase assays were performed with wild-type and kinase dead Snf1 (K84R). The activated kinase was immunoprecipitated from yeast extracts and incubated with GST-Med13^561-650, S608A^ purified from *E. coli. *Serine 608 was mutated to alanine as it potentially is contaminating PAS kinase site (Fig. 5E and 49). In addition, although this site has also been identified as target of PKA mediated phosphorylation 61, we have previously shown that this site does not play a role in Med13 degradation in response to oxidative stress 9. Lastly, it is documented that Snf1 does not phosphorylate GST, so this control was not included here 62. The results (Fig. 5F) show that Snf1 is able to directly phosphorylate degron^571-650^. However, despite taking the precautions listed above, kinase activity was also observed using the kinase dead version. This suggests that another kinase that immunoprecipitates with Snf1 is able to phosphorylate this degron. One strong possibility is Cdk8, which is a proline directed kinase that can phosphorylate the minimal consensus sequence S/T-P 63. Degron^571-650 ^contains one such site (Fig. 5E). Therefore the kinase assays were repeated using Snf1 extracts isolated from a *cdk8*∆ strain. The results show that degron^571-650 ^was phosphorylated by Snf1 and this activity was reduced in the kinase dead control (Fig. 5F). Taken together, these results are suggestive that Snf1 directly phosphorylates Med13^571-650^. However, as some phosphorylation of degron^571-650 ^was observed when Snf1^K84R^ was used, we cannot rule out the possibility that an intermediary kinase may be playing a role.

### Other potential Snf1 sites on Med13 are not needed for its degradation following H_2_O_2_ stress

The Snf1 proteomic screen mentioned above also identified five additional potential Snf1 sites in Med13 57. Intriguingly, these sites all lie within the large IDR of Med13 (Fig. 6A). As IDR’s provide ideal environments for post-translational modifications, which effect signaling events 64, we asked if these additional sites also play a role in Med13 degradation. To address this question, Med13-HA fragments were fused to the SV40 nuclear localization sequence (NLS) and assayed for H_2_O_2_ mediated destruction. The results revealed that the construct that contains both degrons as well as all the potential Snf1 sites (amino acids 306-906) is degraded. However, the Med13^306-570^ construct, that contains the remaining potential Snf1 sites but not the two SCF^Grr1^ responsive degrons, was significantly more stable (Fig. 6B). This suggests that these other potential Snf1 sites do not play a role in the H_2_O_2_-stress mediated degradation of Med13. To address if the head and tail regions of Med13 contain unidentified Snf1 sites, Med13 constructs spanning these regions (amino acids 1-306 and 907-1420) were also tested, as described above, for stability following H_2_O_2_ stress. The results (Fig. 6C) demonstrate that these constructs are not destroyed under these conditions. This supports our conclusion that the Snf1 degron lies within amino acids 571-650. Degron^742-844 ^also contains a potential Snf1 target site (Fig. 6A). However, this degron, when fused to the Gal4 activating domain, is destroyed following H_2_O_2_ stress in wild-type and *snf1*∆ cells alike (Fig. 6D). Taken together, these results support the above conclusions that the Snf1 phosphorylation sites lie within degron^571-650^.

### Snf1 is necessary for cyclin C nuclear release and stress-induced mitochondrial fission

**Figure 7:**
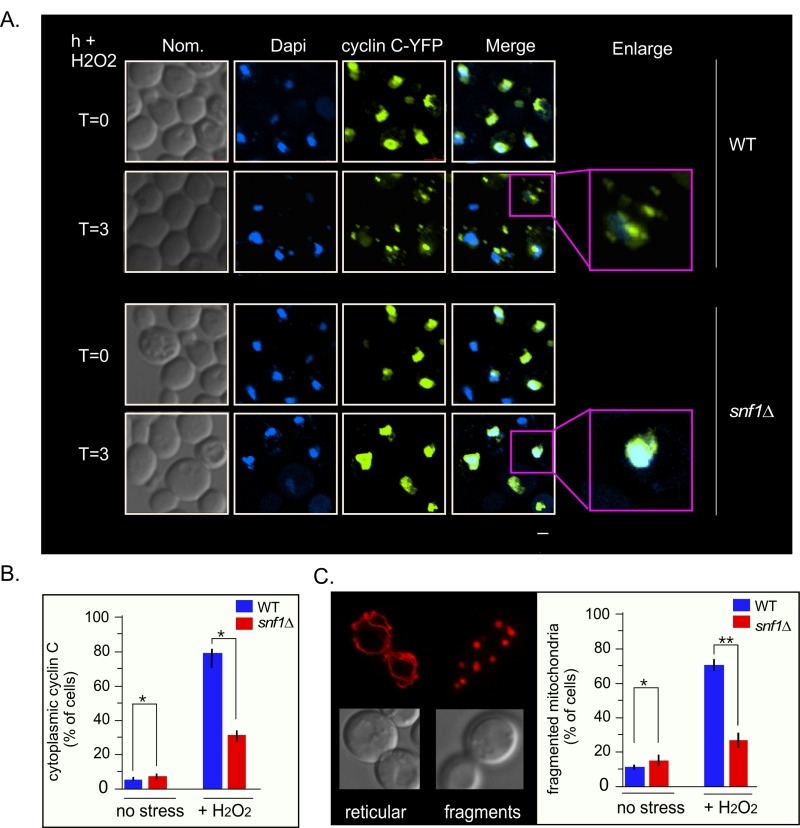
FIGURE 7: Cyclin C remains predominantly nuclear following H_2_O_2_ stress in *snf1*∆*.* **(A)** Fluorescence microscopy of mid-log phase wild-type and *snf1*∆ cells harboring a cyclin C-YFP expression plasmid (pBK38). Cells were stained with Dapi to visualize the nucleus. **(B)** Quantification of the results obtained in (A). At least 200 cells were counted per timepoint from 3 individual isolates. The percent of cells (mean ± SEM) within the population displaying cytoplasmic cyclin C is given. * p < 0.05 difference from wild type. **(C)** Right panel: representative images of the two mitochondrial morphologies scored. Left panel: as in (B) except that percent of cells displaying fragmented mitochondria was scored. Representative images of the mitochondrial morphologies scored are shown in the left hand panel. The percent of cells (mean ± s.e.m.) within the population displaying fragmented mitochondria is given. * p < 0.05 difference from wild type. ** p < 0.01 difference from wild type. Bar = 13 µM.

As Med13 degradation is required for cyclin C nuclear release 9,27, we next tested if Snf1 was also required for this event. To address this, the location of a functional cyclin C-YFP reporter protein 5 before and after stress in wild-type and *snf1*∆ cells was examined (Fig. 7A and quantified in 7B). The results show that a majority of cyclin C-YFP is cytoplasmic in wild-type cells, whereas it remains significantly more nuclear in *snf1*∆ cells (Fig. 7A and quantified in 7B). These data indicate that Snf1 is required for the oxidative stress induced nuclear release of cyclin C. As cyclin C nuclear release initiates stress-induced mitochondrial fission 4,7,8, mitochondrial morphology in *snf1*∆ cells was examined. As previously reported, unstressed wild-type cells exhibited mainly reticular mitochondrial morphology (Fig. 7C) that switched to a predominantly fragmented phenotype after 3 hours of 0.4 mM H_2_O_2_ treatment. In *snf1*∆ cells, significantly less fragmented mitochondria are seen after H_2_O_2_ treatment. Taken together, these results indicate that activation of Snf1 is required for stress-induced mitochondrial fragmentation through cyclin C nuclear release.

### Med13-degron^571-650^ is not required for the degradation of Med13 following H_2_O_2 _stress

**Figure 8:**
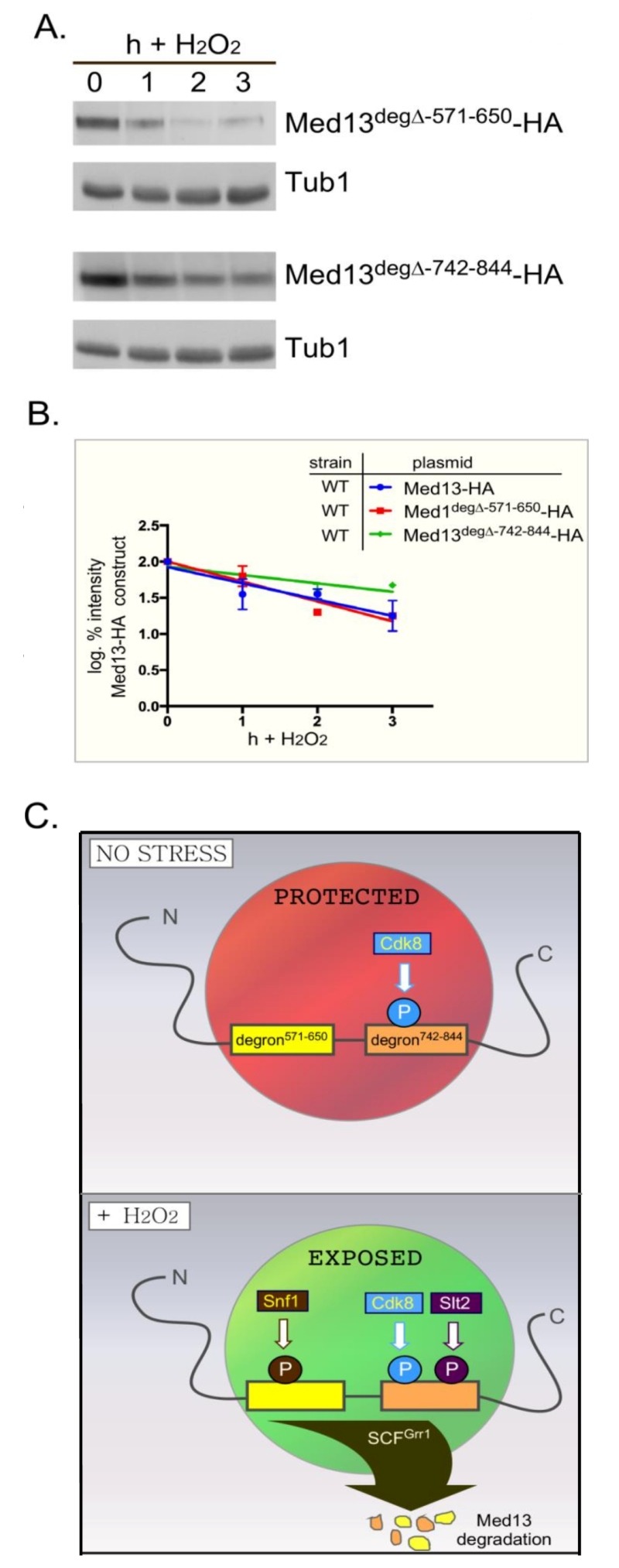
FIGURE 8: Either Med13 degron is sufficient for Med13 degradation. **(A)** *med13*∆ (RSY1701) cells harboring either Med13^571-650deg∆-^HA (pKC805, upper panels) or Med13^742-844deg∆-^HA plasmids (pKC814, lower panels) were treated with 0.4 mM H_2_O_2_ for the timepoints indicated and Med13^deg∆-^HA levels analyzed by Western blot. Tub1 levels were used as a loading control. **(B)** Degradation kinetics of the results shown in (A). Values represent averages ± SD from a total of at least two Western blots from independent experiments. For clarity, the degradation kinetics of wild-type Med13-HA from previous experiments was included. **(C) **Model depicting how two SCF^Grr1^ phospho-degrons mediate the destruction of Med13 following H_2_O_2_ stress. In unstressed cells cyclin C-Cdk8 phosphorylates degron^742-844^ 9 but both degrons are protected by an unknown mechanism from Snf1 and Slt2 kinase activity (depicted by the red circle). Following H_2_O_2_ stress Snf1 and Slt2 are activated and permitted access to the now exposed degrons. This results in SCF^Grr1^ mediated degradation of Med13 and cyclin C nuclear release (not shown).

We next addressed if Snf1 mediated phosphorylation of Med13 is required for Med13 degradation. Degron^571-650 ^was deleted from full length Med13 and the degradation kinetics of this construct (Med13^deg∆571-650^) was examined in *med13*∆ cells. The results show that Med13^deg∆571-650^ was degraded with kinetics similar to wild type (Fig. 8A upper two panels, quantified in Fig. 8B). We also observed that this construct retained cyclin C in the nucleus in unstressed cells and released it into the cytoplasm following H_2_O_2_ stress (Fig. S5). These results were unexpected as deletion or inactivation of Snf1 results in Med13 stabilization following H_2_O_2 _stress (Fig. 3). Similarly, deletion of the Slt2 responsive degron (amino acids 742-844) only partially rescued the deletion (Fig. 8A, bottom two panels) whereas Med13 is significantly stabilized in *slt2*∆ cells following H_2_O_2_ stress 9. These results suggest a complex model in which the function of either Snf1 or Slt2 is only required when their respective degron is present. If correct such a model would predict that the degron impeaches degradation when not phosphorylated. Moreover, these studies predict independent roles for these two signaling pathways that ultimately direct in the same outcome of Med13 degradation.

## DISCUSSION

Our previous work has shown that nuclear release of the yeast and mammalian cyclin C predisposes cells to initiate PCD following stress 7,8. In *S. cerevisiae,* this nuclear release requires the destruction of Med13 27, mediated by the E3 ligase complex SCF^Grr1^, which requires Slt2 and Cdk8 activity 9. In this current work, we provide evidence that Sak1 activated Snf1 is also required for H_2_O_2_ induced Med13 degradation and cyclin C nuclear release. In the absence of this kinase, Med13 degradation following H_2_O_2_ stress is inhibited and cyclin C remains predominantly nuclear. Consistent with this model, we showed that Snf1 is required for the degradation of the degron^571-650^. Paradoxically, deletion of this degron did not prevent H_2_O_2_ induced destruction of Med13. Instead, this mutant exhibited H_2_O_2_ induced Med13 degradation and cyclin C nuclear release. Intriguingly, deletion of the Slt2-resonsive degron (Med13^742-844^) also did not protect Med13 from H_2_O_2_ induced degradation, although the protein was slightly more stable than wild type. Taken together, this study indicates that Slt2 and Snf1 pathways cooperate to trigger Med13 destruction. The use of two required pathways may help insure the proper signals are in place to target Med13.

To accommodate these two results, the following model that best fits the data is proposed (outlined in Fig. 8C). In unstressed cells the IDR of Med13, which encompasses both degrons, is protected from SCF^Grr1^ activity (depicted by red circle in Fig. 8C) by an unknown mechanism. Following H_2_O_2_ stress two events happen, the hierarchy of which is unknown. The protection is lost (depicted by green circle in Fig. 8C) and activated Snf1 and Slt2 phosphorylate their respective degrons, triggering SCF^Grr1^ mediated degradation of Med13. Although we currently favor the hypothesis that Snf1 directly phosphorylates Med13, we could not definitively exclude the possibility that Snf1 may promote Med13 phosphorylation indirectly via an intermediary kinase. That being said, the model presented in Fig. 8C could accommodate this possibility. More importantly however, the model supports the observation that both the AMPK and MAPK pathways act independently of each other (Fig. 4), and are required for Med13 degradation. However, in the absence of either the Snf1 or Slt2 degron, this protection is lost, allowing SCF^Grr1^ to recognize either activated degron. This model also accounts for the observation that when either degron is expressed in isolation, it requires its respective kinase to render it recognizable by SCF^Grr1^.

If this model is correct, then how could this region be protected from AMPK and MAPK activity in unstressed cells? One strong possibility could be connected to the fact that both degrons lie within the very large IDR of Med13 (Fig. 2A). IDR’s are known to endow proteins with highly malleable structures that undergo disorder-to-order transitions 65. It has also been proposed that IDR’s can have different binding partners that transiently associate 66. In some cases, this can lead to proteins binding the same region that possess unrelated, or even opposite functions 67. Thus, proteins that contain IDR’s are frequently involved in signaling as they can easily change their conformational state in response to changing environmental conditions. Taken together, it is feasible to propose that in unstressed cells, the IDR of Med13 is in one conformational state that associates with an unknown protein, conferring protection to this region. Upon stress, the confirmation changes and the degron becomes exposed. This model is also consistent with the observation that IDR’s are notorious for being regulated by multiple kinases 64,68. This has led to the idea that IDR’s and multiple phosphorylation events together provide structural variability 69, resulting in ultra-sensitive molecular switches that are triggered at a threshold level of phosphorylation. Thus, our results suggest a model in which Med13 degradation is regulated by three types of different kinases, a cyclin dependent kinase, a MAPK and an AMPK.

Lastly, here we show that Snf1 phosphorylates Med13, either directly or by an intermediary kinase, as well as associating with the CKM before stress (Fig. 5D). These results are consistent with a growing number of papers that have shown that a sub-population of the Snf1-Gal3 isoform is present in the nucleus under normal conditions 52,60,70, as well as enriched in the nucleus upon glucose starvation 34,35,36. In addition, Sak1 is needed for Snf1 nuclear localization 71. In support of this model, Snf1, Gal83 and Sak1 localize to the *SUC2* promotor under non-starvation conditions 52. This promotor is also negatively regulated by the CKM 72. Repression is relieved by Snf1 mediated phosphorylation of two proteins known to repress *SUC2* expression, the DNA binding protein Mig1 73 and the glucose kinase Hxk2 52,74, causing them to be released into the cytoplasm 75,76. Likewise, carbon starvation results in the degradation of cyclin C 2, one event that occurs in the cytoplasm 4. Surprisingly, we found that although Sak1 is needed for Med13 destruction following H_2_O_2_ stress, deletion of Gal83 has no effect (Fig. 3A and S2A). However, deletion of all three β-subunits does inhibit Med13 degradation. This would suggest that in the absence of Gal83, either Sip1 or Sip2 are able to activate nuclear Snf1. However, the Snf1-Sip1 and Snf1-Sip2 isoforms have not been reported to be nuclear, dispersing either to the vacuolar membrane (Sip1) or remaining cytoplasmic following carbon deprivation (Sip2) 34,35,36,77. Further studies need to be executed to address if Sip1 or Sip2 can translocate into the nucleus in the absence of Gal83. Intriguingly, just recently the Mitochondrial Voltage-Dependent Anion Channel Protein Por1 (yVDAC1) has been shown to enhance Snf1 nuclear enrichment by promoting the nuclear enrichment of Gal83 70. This unexpected finding serves to emphasize that there is still much to learn about the role Snf1 kinase plays in response to changing environmental conditions.

## MATERIALS AND METHODS

### Yeast strains and plasmids 

All strains used in this study are listed in Table S1. Most experiments were performed in the *S. cerevisiae* W303 strain 78 and are listed in Table S1. Exceptions to this are the *sak1*∆ (YPDahl17, a gift from S. Hohmann 79), the *sip1*∆* sip2*∆ (MML1445, a gift from E. Herrero) and *psk1*∆* psk2*∆ strains which are W303-1A strains. The *sip1*∆* sip2*∆* gal83*∆ strain (MSY557, a gift from M. Schmidt 80) is an S288c strain. Finally, the Y2H assays were performed in PJ69-4a 40 that was obtained from the Yeast Resource center, a gift from S. Fields. In accordance with the Mediator nomenclature unification effort 81, we use *CNC1* and *CDK8* gene designations for cyclin C (*SSN8*/*UME3*/*SRB11*) and Cdk8 (*SSN3*/*UME5*/*SRB10*) respectively. The *snf1*∆ and *gal83*∆ strains (RSY1949 and RSY2080 respectively) were constructed using gene replacement methodology as described 82. The *sak1*∆ (RSY1976) was a gift from S. Hohmann 79. All cells were grown at 30°C.

All plasmids used in this study are listed in Table S2. The wild-type epitope tagged plasmids pKC801, pKC803 (*MED13-*HA), pBK38 (*ADH1_Pro_*-*CNC1*-YFP), Snf1-Myc and *ADH1_Pro_*-*CNC1*-MYC (pKC337) are functional and have been previously described 1,4,5,9,83,84. All other plasmids were constructed using PCR cloning techniques and details are available upon request. In short, all constructs were amplified from plasmid DNA using Phusion Taq (Thermo), digested using Thermo fast digest restriction enzymes and ligated using Thermo fast ligase into their respective vectors. Site directed mutagenesis (New England Bio-Labs Q5) was used to create plasmids harboring amino acid mutations and the change was confirmed by sequencing (Eurofins Genomics). The *MED13* Y2H plasmids were constructed by PCR cloning regions of Med13 in frame with the Gal4 activating domain of pACT2. The NLS-Med13 fusion constructs were made by first creating a backbone vector (pNLS-HA) that contains the SV40 nuclear localization sequence (NLS) in frame with a single HA epitope tag under the control of the *ADH1* promotor. PCR-cloning was then used to place fragments of Med13 in frame with NLS. All in frame fusion proteins were verified by sequence analysis. Other plasmids that were used in this study that have been previously described.

### Cell growth 

Yeast cells were grown in either rich, non-selective medium (YPDA) or synthetic minimal medium (SC) allowing plasmid selection as previously described 1. For all experiments, the cells were grown to mid-log phase (~ 6x10^6^ cells/ml) before treatment with low concentrations of 0.4 mM H_2_O_2 _as previously described 5. 25 ml of cells were collected per timepoint, washed in water, then the pellet flash frozen in liquid nitrogen. Yeast two hybrid experiments were executed as described 41. *E. coli *cells were grown in LB medium with selective antibiotics. For the kinase assays cells were initially grown in SD-Ura (1.7 g Yeast Nitrogen Base, 5 g Ammonium Sulfate, 20 g Dextrose per liter), overnight, diluted 1:100-fold into 500 ml of SD-Ura, and grown for 10-12 h. Thereafter the cells were pelleted, and resuspended in 500 ml SGal-Ura (1.7 g Yeast Nitrogen Base, 5 g Ammonium Sulfate, 20 g Galactose per liter)

### Western blot analysis and co-immunoprecipitation

Tagged full length Med13-HA constructs were detected by using NaOH lysis of cell pellets exactly as described in 9. To detect Med13-HA, 1 in 5000 dilutions of anti-HA antibodies (Abcam) were used. The co-immunoprecipitation analysis was performed essentially as described 85. Anti-alpha tubulin antibodies (12G10) were obtained from the Developmental Studies Hybridoma Bank, University of Iowa. Western blot signals were detected using either goat anti-mouse or goat anti-rabbit secondary antibodies conjugated to alkaline phosphatase (Sigma) and the CDP-Star chemiluminescence kit (Thermo). Signals were quantitated by CCD camera imaging (Kodak) and standardized to the loading control. All degradation assays were performed two or three times. SEM’s were generated for each point (error bars are indicated on the graphs) and the data analyzed using linear regression analysis using GraphPad Prism 7 program.

### Snf1 activation and kinase assays

The Snf1 activation assays were executed as follows. Cells were grown to mid-log phase and treated with 0.4 mM H_2_O_2_ for the times indicated They were immediately boiled for 3 minutes and pellets frozen using liquid nitrogen. Protein extracts were made using the NaOH method described in 9 and analyzed by SDS-PAGE and Western blotting. Anti-phospho-Thr172-AMPK (Cell Signaling Technology; Cat. No. 2531) was used to detect phosphorylated Snf1 and polyhistidine antibody H1029 (Sigma-Aldrich) to detect Snf1. This is possible as Snf1 ORF contains 13 contiguous histidine residues at its N terminus.

The *in vitro* kinase assays were executed as follows. Wild-type or *cdk8*∆ cells harboring either myc-tagged Snf1 or Snf1 kinase dead (K84R) under the control of the *GAL1-10* promotor, were first grown in SD-Ura followed by SGal-Ura as described above and then resuspended in lysis buffer (20 mM HEPES, 10 mM KCl, 1 mM EDTA, 1 mM ethylene glycol tetra-acetic acid (EGTA), 50 mM NaCl, 10% glycerol, 1 mM β-mercaptoethanol, Pierce™ Protease Inhibitor Tablets, pH 7.4 with phosphatase inhibitors). Resuspended cultures were lysed using a Microfluidics M-110P homogenizer (Microfluidics) and cell debris removed by centrifugation at 12,000 x g for 30 min. Supernatants were incubated with 5-10 µl of Myc-conjugated magnetic beads (Cell Signaling) for 2-3 h at 4°C. Beads were separated using magnetic force and washed four times with 1 ml of lysis buffer lacking protease inhibitors. Beads containing Myc-tagged proteins were used directly for *in vitro* kinase assays without eluting. GST-Med13^571-650S608A^ was purified from *E. coli *BL21 DE3 strain as previously described 9 and incubated with the Snf1 immunoprecipitates in 30 μl of reaction buffer containing 1X Snf1 kinase buffer (50 mM Tris-HCl, 10 mM MgCl_2_, 1 mM dithiothreitol (DTT), pH 7.5, 10 μM ATP, and 7.5 μCi of γ32P-ATP, and reactions were incubated for 35 min at 30°C. Kinase assays were stopped by the addition of SDS-PAGE sample buffer, analyzed by SDS-PAGE, stained with coommassie (30 min), destained for 2 h, dried and exposed to film.

### Fluorescence Microscopy

Cyclin C-YFP subcellular localization and mitochondrial morphology were monitored as described previously 4,7. For all experiments, the cells were grown to mid-log (6x10^6^ cells/ml), treated with 0.4 mM H_2_O_2_ for the timepoints indicated, then analyzed by fluorescence microscopy. Cyclin C-YFP release analysis was performed on cells stained with DAPI in mounting medium (10 mg/ml p-phenylenediamine, 50 ng/ml 4',6-diamidino-2-phenylindole, Molecular Probes) to visualize nuclei and prevent photo bleaching. Images were obtained using a Nikon microscope (model E800) with a 100X objective with 1.2X camera magnification (Plan Fluor Oil, NA 1.3) and a CCD camera (Hamamatsu model C4742). Data were collected using NIS software and processed using Image Pro software. All images of individual cells were optically sectioned (0.2 µM slices at 0.3 µM spacing) deconvolved and the slices were collapsed to visualize the entire fluorescent signal within the cell. Cyclin C-YFP foci were scored as being cytoplasmic when 3 or more foci were observed outside of the nucleus. Mitochondrial fission assays were performed on live cells as described 7. In brief, mitochondrial fission was scored positive if no reticular mitochondria were observed that transversed half the cell diameter. Fusion was scored when cells exhibited one or more reticular mitochondria the diameter of the cell. Fission and fusion was scored for 200 cells from three independent isolates. Statistical analysis was performed using the Student’s T-test.

## SUPPLEMENTAL MATERIAL

Click here for supplemental data file.

All supplemental data for this article are also available online at http://microbialcell.com/researcharticles/snf1-cooperates-with-the-cwi-mapk-pathway-to-mediate-the-degradation-of-med13-following-oxidative-stress/.
