# Supplementary material for: Snf1 cooperates with the CWI MAPK pathway to mediate the degradation of Med13 following oxidative stress
Source: Microb Cell. 2018 Jun 25;5(8):357–70. doi: 10.15698/mic2018.08.641 (PMC6116281; doi:10.15698/mic2018.08.641)
Supplement: Supplementary file 1 [file mic-05-357-s01.pdf]

Supplemental Table 1. Yeast strains used in this study.

Strain	Genotype*	Source
RSY10		[1]
RSY1696	*cnc1::KANMX6*	[2]
RSY1701	*med13::HIS3*	[3]
RSY1707	*mid2::HIS3 mtl1::TRP1 wsc1::KANMX4*	[4]
RSY1770	*grr1Δ::his5$^+$*	[5]
RSY1726	*cdk8Δ::KANMX4*	[3]
RSY1949	*gal83::KANMX4*	This study
RSY2080	*snf1::KANMX4*	This study
YPDahl17	*sak1::KANMX4*	[6]
MML1445	*sip1::natMX4 sip2:: KANMX4*	[7]
MSY557	*sip1::HIS3 sip2:: HIS3 gal83::HIS3*	[8]
JGY1		[9]
JGY4	*psk1::HIS3 psk2Δ:: KANMX4*	[10]
PJ69-4	*LYS2::GAL1-HIS3 GAL2-ADE2 met2::GAL7-lacZ gal4Δ gal80Δ*	[11]

Genotype of all strains is *MAT***a** *ade2 ade6 can1-100 his3-11,15 leu2-3,112 trp1-1 ura3-1* except YPDahl17, MML1445, JGY1 and JGY4 which are *MAT***a** *ade2-1 can1-100 his3-11,15 leu2-3,112 trp1-1 ura3-1 and* PJ69-4 which is *MAT***a** *trp1-901 leu2-3,112 ura3-52 his3-200 gal4Δ gal80Δ.*

Supplemental Table 2. Plasmids used in this study.

Plasmid Name	Gene	Epitope Tag	Marker	Promoter	2µ/ CEN	Reference
pBK38	*CNC1*	YFP	*URA3*	*ADH1*	CEN	[12]
pKC337	*CNC1*	*CNC1*	*TRP1*	*ADH1*	CEN	[13]
pKC801	*MED13*	3HA	*URA3*	*TRP1*	CEN	[5]
pLR166	$CNC1^{S266A}$	*CNC1*	*TRP1*	*TRP1*	CEN	[14]
pKC803	*MED13*	3HA	*LEU2*	*ADH1*	CEN	[5]
pKC805	$MED13^{571\text{-}650deg\Delta}$	3HA	*URA3*	*ADH1*	CEN	This study
pKC814	$MED13^{742\text{-}844deg\Delta}$	3HA	*URA3*	*ADH1*	CEN	This study
pDS8	$GAL4AD\text{-}MED13^{571\text{-}906}$	1HA	*LEU2*	*ADH1*	2µ	[5]
pDS15	$GAL4AD\text{-}MED13^{571\text{-}650}$	1HA	*LEU2*	*ADH1*	2µ	[5]
pDS16	$GAL4AD\text{-}MED13^{651\text{-}906}$	1HA	*LEU2*	*ADH1*	2µ	[5]
pDS32	$GAL4AD\text{-}MED13^{742\text{-}844}$	1HA	*LEU2*	*ADH1*	2µ	[5]
pDS44	$GAL4AD\text{-}MED13^{571\text{-}650\ S636}$	1HA	*LEU2*	*ADH1*	2µ	This study
pDS51	$GAL4AD\text{-}MED13^{571\text{-}650\ S636,\ S634A}$	1HA	*LEU2*	*ADH1*	2µ	This study
pDS55	$GAL4AD\text{-}MED13^{571\text{-}650}$ *S587A*	1HA	*LEU2*	*ADH1*	2µ	This study
pDS56	$GAL4AD\text{-}MED13^{571\text{-}650}$ *S58A7, S636A, S634A*	1HA	*LEU2*	*ADH1*	2µ	This study
pDS45	$NLS\text{-}Med13^{1\text{-}306}$	1HA	*LEU2*	*ADH1*	2µ	This study
pDS46	$NLS\text{-}Med13^{306\text{-}570}$	1HA	*LEU2*	*ADH1*	2µ	This study
pDS47	$NLS\text{-}Med13^{907\text{-}1420}$	1HA	*LEU2*	*ADH1*	2µ	This study
pDS52	$NLS\text{-}Med13^{307\text{-}570}$	1HA	*LEU2*	*ADH1*	2µ	This study
pDS54	$GST\text{-}Med13^{571\text{-}650,S608A}$	GST	*AMP*	-	-	This study
pJG1215	HIS_6*-PSK1-KD*	HIS_6	*AMP*	-	-	[10]
pUM504	*CDK8*	1HA	*TRP*	*GPD*	CEN	[15]
pACT2	*GAL4AD*	1HA	*LEU2*	*ADH1*	2µ	[16]
pAS2	*GAL4BD*	1HA	*TRP*	*ADH1*	2µ	[16]
pAS2-Grr1	*GAL4BD-GRR1*	1HA	*TRP*	*ADH1*	2µ	[17]
pAS2-Grr1ΔLΔF	*GAL4BD-GRR1Grr1ΔLΔF*	1HA	*TRP*	*ADH1*	2µ	[17]
pJG1465	$GAL4AD\text{-}MED13^{504\text{-}703}$	1HA	*TRP*	*ADH1*	2µ	[9]
pAS2-Psk1	*GAL4BD-PSK1*	1HA	*TRP*	*ADH1*	2µ	[9]
JG1193	Snf1	8Myc	*URA3*	*Snf1*	CEN	[18]
JG1338	$Snf1^{K84R}$	8Myc	*URA3*	*Snf1*	CEN	[19]
	Snf1	8Myc	*URA3*	*GAL1-10*	2µ	[19]
	$Snf1^{K84R}$	8Myc	*URA3*	*GAL1-10*	2µ	[19]
pNLS-HA	SV40 NLS	1HA	*LEU2*	*ADH1*	2µ	This study
Mt-Cherry	Mito-targeting	mCherry	*TRP1*	*ADH1*	CEN	This study
pRS314	-	-	*TRP1*	-	CEN	[20]
pRS316	-	-	*URA*	-	CEN	[20]

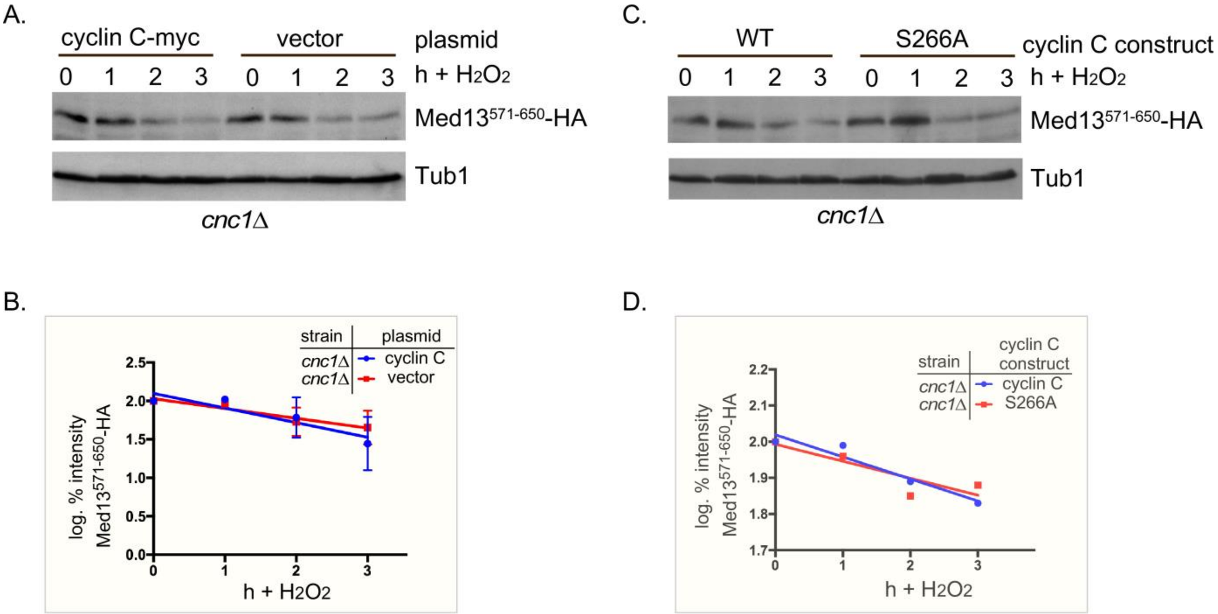

FIGURE S1: Phosphorylation of cyclin C is not required for degradation of Med13$^{571\text{-}650}$. **(A)** *cnc1*Δ cells (RSY1696) harboring degron$^{571\text{-}650}$ (pDS15) and either wild-type cyclin C (pKC337) or a vector control (pRS314) were treated with 0.4 mM H_2O_2 for the timepoints indicated and Med13$^{571\text{-}650}$-HA levels analyzed by Western blot. Tub1 levels were used as loading controls. **(B)** Degradation kinetics of the degron$^{571\text{-}650}$ constructs shown in (A). Values represent averages ± SD from a total of at least two Western blots from independent experiments. **(C)** As in (A) except that *cnc1*Δ harboring either wild-type cyclin C (pKC337) or a phospho-mutant (cyclin C S266A, pLR166) was examined. **(D)** Degradation kinetics of results shown in (C).

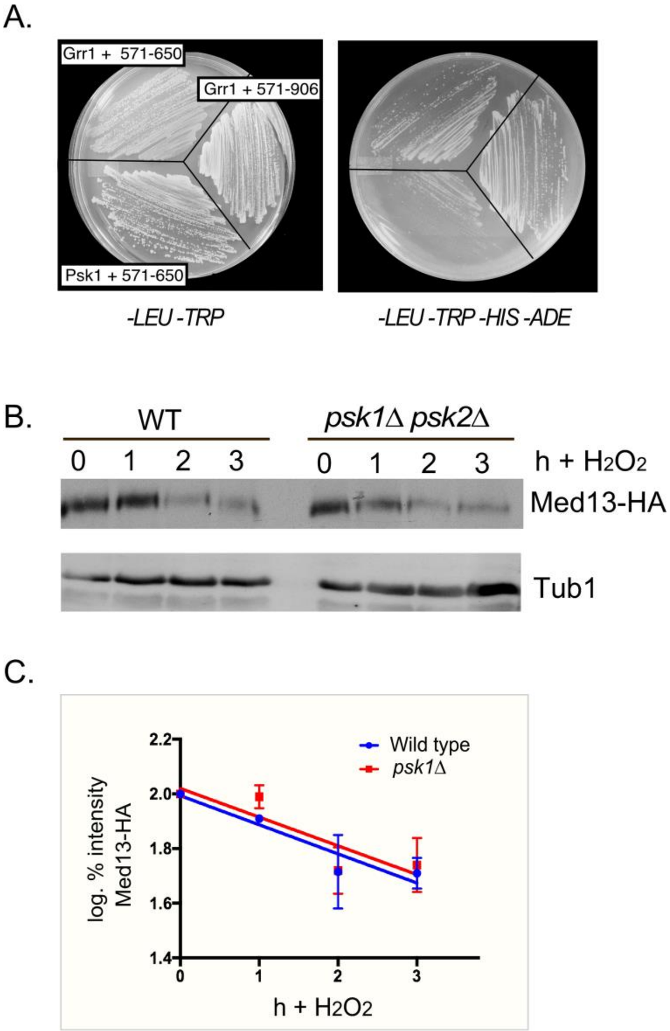

FIGURE S2: The PAS kinase can associate with Med13 but is not required for its H_2O_2 mediated degradation. (A) Y2H analysis of cells harboring the Med13 construct shown with either Grr1 or Psk1. PJ69-4 cells harboring the Med13-activating domain constructs shown and either pAS-Grr1 or pAS2-Psk1 which has previously been shown to interact with Med13$^{505\text{-}703}$ [9]. The cells were grown on *-LEU, -TRP* drop out medium to select for both plasmids (left panel) or *-TRP, -LEU, -HIS –ADE* (right panel) to test for Med13-Grr1 interaction. **(B)** Wild-type (RSY10) and *psk1*Δ *psk2*Δ (JGY4) cells harboring Med13-HA (pKC801) were treated with 0.4 mM H_2O_2 for the timepoints indicated and Med13 levels analyzed by Western blot. Tub1 levels were used as loading controls. **(C)** Degradation kinetics of the results shown in (B). Values represent averages ± SD from a total of at least two Western blots from independent experiments.

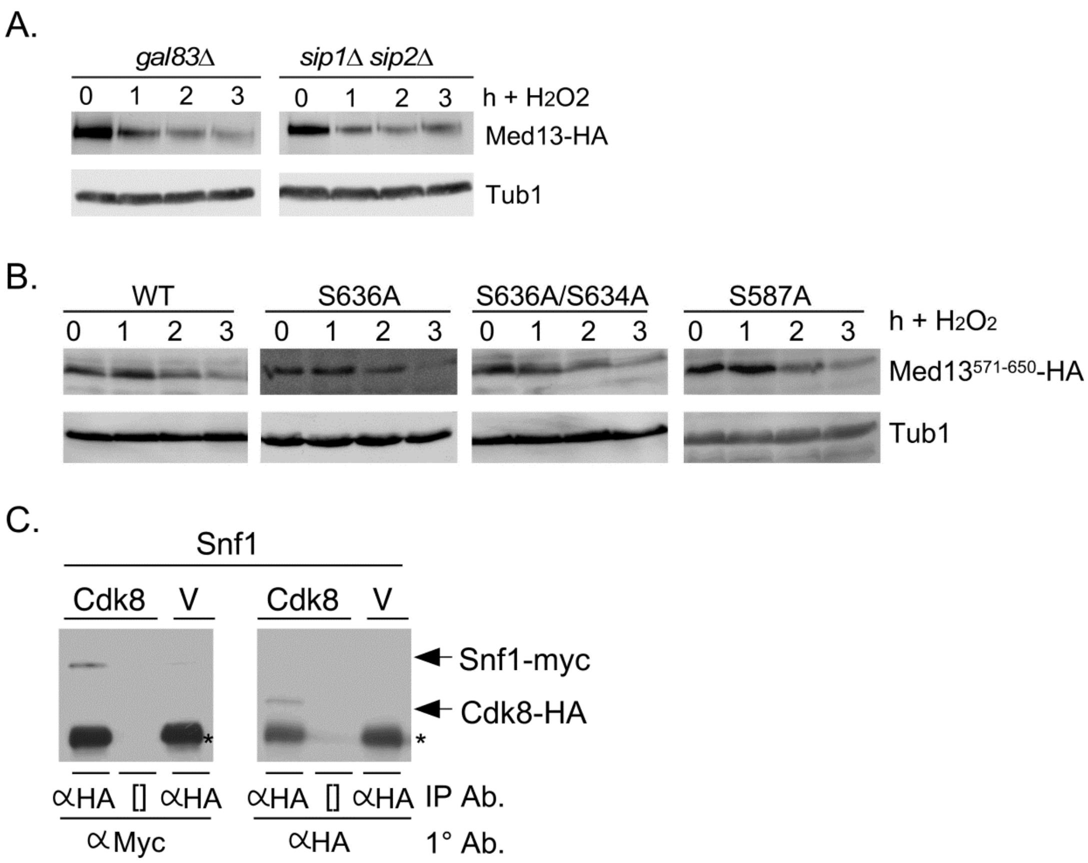

FIGURE S3: **(A)** Cells with the genotypes shown harboring Med13-HA (pKC801) were treated with 0.4 mM H_2O_2 for the timepoints indicated and Med13 levels analyzed by Western blot. Tub1 levels were used as loading controls. **(B)** Wild-type (RSY10) cells harboring either degron$^{571\text{-}650}$ (pDS15) or with the mutations shown were treated with 0.4 mM H_2O_2 for the timepoints indicated and analyzed by Western blot. Tub1 levels were used as loading controls. **(C)** Control experiment for the co-immunoprecipitation analysis shown in Fig. 5D showing that cells harboring a vector control are unable to pull down Snf1-myc whereas Cdk8-HA can.

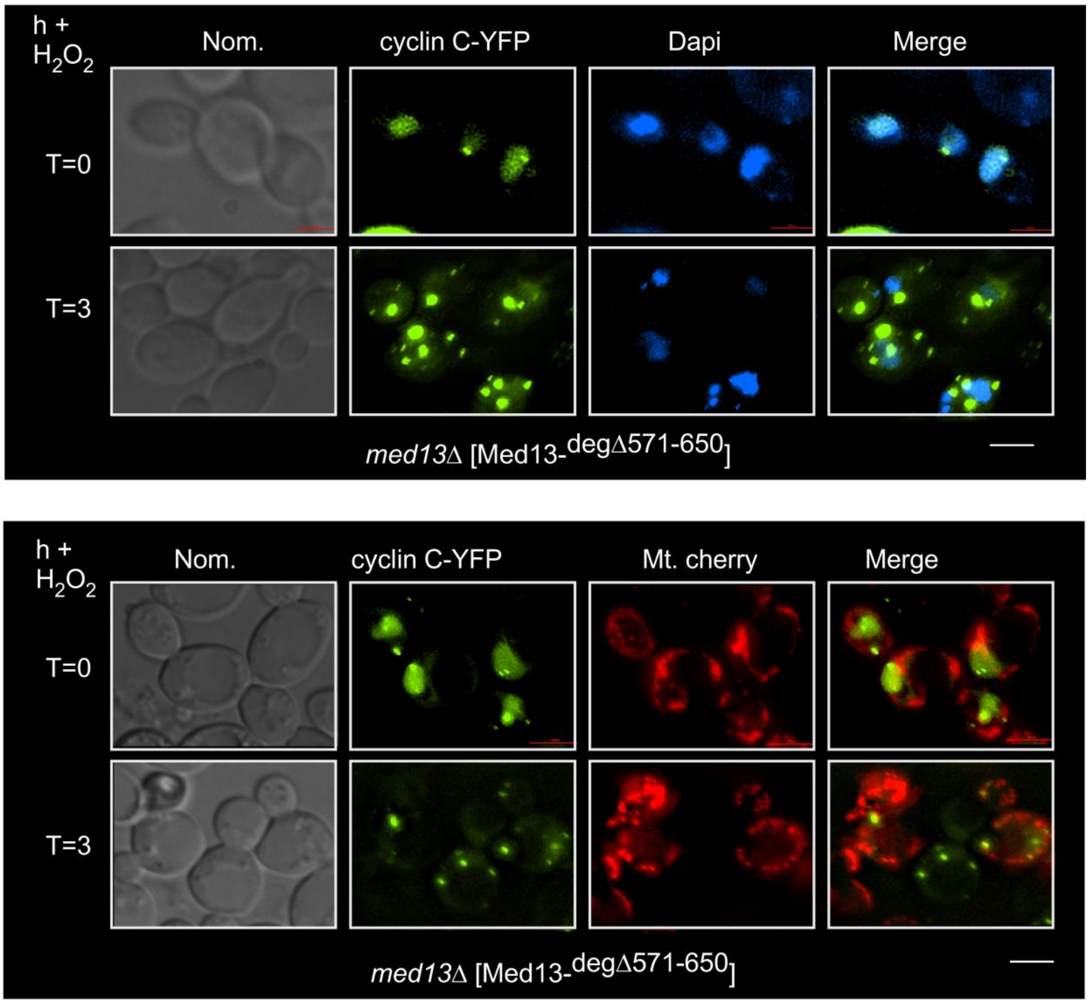

FIGURE S4. Upper panel: cyclin C is released from the nucleus following H_2O_2 stress in *med13*Δ cells harboring Med13$^{571\text{-}650deg\Delta\text{-}}$HA (pKC805) as the only source of Med13. Fluorescence microscopy of mid-log phase *med13*Δ harboring pKC805 and cyclin C-YFP (pBK38) were analyzed as shown before and after 0.4 mM H_2O_2 stress. Cells were stained with Dapi to visualize the nucleus. Lower Panel: as in upper panel except that the mitochondrial marker mCherry was also expressed. Bar = 13 µM.

Supplemental References

1. Strich R, Slater MR, Esposito RE (**1989**). Identification of negative regulatory genes that govern the expression of early meiotic genes in yeast. **Proc. Natl. Acad. Sci. USA** 86(10018-10022. doi: 10.1073/pnas.86.24.10018

2. Cooper KF, Mallory MJ, Egeland DB, Jarnik M, Strich R (**2000**). Ama1p is a meiosis-specific regulator of the anaphase promoting complex/cyclosome in yeast. **Proc Natl Acad Sci U S A** 97(26): 14548-14553. doi: 10.1073/pnas.250351297

3. Khakhina S, Cooper KF, Strich R (**2014**). Med13p prevents mitochondrial fission and programmed cell death in yeast through nuclear retention of cyclin C. **Mol Biol Cell** 25(18): 2807-2816. doi: 10.1091/mbc.E14-05-0953

4. Jin C, Parshin AV, Daly I, Strich R, Cooper KF (**2013**). The cell wall sensors Mtl1, Wsc1, and Mid2 are required for stress-induced nuclear to cytoplasmic translocation of cyclin C and programmed cell death in yeast. **Oxid Med Cell Longev** 2013(320823. doi: 10.1155/2013/320823

5. Stieg DC, Willis SD, Ganesan V, Ong KL, Scuorzo J, Song M, Grose J, Strich R, Cooper KF (**2018**). A complex molecular switch directs stress-induced cyclin C nuclear release through SCF(Grr1)-mediated degradation of Med13. **Mol Biol Cell** 29(3): 363-375. doi: 10.1091/mbc.E17-08-0493

6. Ye T, Elbing K, Hohmann S (**2008**). The pathway by which the yeast protein kinase Snf1p controls acquisition of sodium tolerance is different from that mediating glucose regulation. **Microbiology** 154(Pt 9): 2814-2826. doi: 10.1099/mic.0.2008/020149-0

7. Perez-Sampietro M, Casas C, Herrero E (**2013**). The AMPK family member Snf1 protects Saccharomyces cerevisiae cells upon glutathione oxidation. **PLoS One** 8(3): e58283. doi: 10.1371/journal.pone.0058283

8. Ye T, Elbing K, Hohmann S (**2008**). The pathway by which the yeast protein kinase Snf1p controls acquisition of sodium tolerance is different from that mediating glucose regulation. **Microbiology** 154(Pt 9): 2814-2826. doi: 10.1099/mic.0.2008/020149-0

9. DeMille D, Bikman BT, Mathis AD, Prince JT, Mackay JT, Sowa SW, Hall TD, Grose JH (**2014**). A comprehensive protein-protein interactome for yeast PAS kinase 1 reveals direct inhibition of respiration through the phosphorylation of Cbf1. **Mol Biol Cell** 25(14): 2199-2215. doi: 10.1091/mbc.E13-10-0631

9. DeMille D, Bikman BT, Mathis AD, Prince JT, Mackay JT, Sowa SW, Hall TD, Grose JH (**2014**). A comprehensive protein-protein interactome for yeast PAS kinase 1 reveals direct inhibition of respiration through the phosphorylation of Cbf1. **Mol Biol Cell** 25(14): 2199-2215. doi: 10.1091/mbc.E13-10-0631

10. DeMille D, Badal BD, Evans JB, Mathis AD, Anderson JF, Grose JH (**2015**). PAS kinase is activated by direct SNF1-dependent phosphorylation and mediates inhibition of TORC1 through the phosphorylation and activation of Pbp1. **Mol Biol Cell** 26(3): 569-582. doi: 10.1091/mbc.E14-06-1088

11. James P, Halladay J, Craig EA (**1996**). Genomic libraries and a host strain designed for highly efficient two-hybrid selection in yeast. **Genetics** 144(4): 1425-1436. PMID: 8978031

12. Cooper KF, Scarnati MS, Krasley E, Mallory MJ, Jin C, Law MJ, Strich R (**2012**). Oxidative-stress-induced nuclear to cytoplasmic relocalization is required for Not4-dependent cyclin C destruction. **J Cell Sci** 125(Pt 4): 1015-1026. doi: 10.1242/jcs.096479

13. Cooper KF, Mallory MJ, Smith JB, Strich R (**1997**). Stress and developmental regulation of the yeast C-type cyclin Ume3p (Srb11p/Ssn8p). **EMBO J** 16(15): 4665-4675. doi: 10.1093/emboj/16.15.4665

14. Jin C, Strich R, Cooper KF (**2014**). Slt2p phosphorylation induces cyclin C nuclear-to-cytoplasmic translocation in response to oxidative stress. **Mol Biol Cell** 25(8): 1396-1407. doi: 10.1091/mbc.E13-09-0550

15. Cooper KF, Mallory MJ, Strich R (**1999**). Oxidative stress-induced destruction of the yeast C-type cyclin Ume3p requires phosphatidylinositol-specific phospholipase C and the 26S proteasome. **Mol Cell Biol** 19(5): 3338-3348. doi: 10.1128/mcb.19.5.3338

16. Van Criekinge W, Beyaert R (**1999**). Yeast Two-Hybrid: State of the Art. **Biol Proced Online** 2(1-38. doi: 10.1251/bpo16

17. Wang R, Solomon MJ (**2012**). Identification of She3 as an SCF(Grr1) substrate in budding yeast. **PLoS One** 7(10): e48020. doi: 10.1371/journal.pone.0048020

18. Simpson-Lavy KJ, Johnston M (**2013**). SUMOylation regulates the SNF1 protein kinase. **Proc Natl Acad Sci U S A** 110(43): 17432-17437. doi: 10.1073/pnas.1304839110

19. Strogolova V, Orlova M, Shevade A, Kuchin S (**2012**). Mitochondrial porin Por1 and its homolog Por2 contribute to the positive control of Snf1 protein kinase in Saccharomyces cerevisiae. **Eukaryot Cell** 11(12): 1568-1572. doi: 10.1128/EC.00127-12

20. Sikorski RS, Hieter P (**1989**). A system of shuttle vectors and yeast host strains designed for efficient manipulation of DNA in *Saccharomyces cerevisiae*. **Genet.** 122: 19-27. PMID: 2659436
